# Improvement in Abrasive Wear Resistance of Metal Matrix Composites Used for Diamond–Impregnated Tools by Heat Treatment

**DOI:** 10.3390/ma16186198

**Published:** 2023-09-13

**Authors:** Elżbieta Cygan-Bączek, Sławomir Cygan, Piotr Wyżga, Pavel Novák, Ladislav Lapčák, Andrzej Romański

**Affiliations:** 1 Łukasiewicz Research Network—Krakow Institute of Technology, Zakopiańska 73 Str., 30-418 Krakow, Poland; 2 Department of Metals and Corrosion Engineering, University of Chemistry and Technology Prague, Technická 5, 166 28 Prague, Czech Republic; 3 Central Laboratories, University of Chemistry and Technology Prague, Technicka 5, 166 28 Prague, Czech Republic; 4 Faculty of Metals Engineering and Industrial Computer Science, AGH University of Science and Technology, 30 Mickiewicz Avenue, 30-059 Krakow, Poland; aromansk@agh.edu.pl

**Keywords:** matrix, diamond-impregnated tools, ball milling, heat treatment, spark plasma sintering, abrasive wear resistance

## Abstract

This work presents the possibilities of producing a substitute for a commercial matrix material for sintered metal–diamond tools which is characterized by increased tribological properties required in machining natural stones and concrete. In this study, the improvement in wear behavior of sintered pre-alloyed matrix caused by a thermal treatment was investigated. Several mixtures made of commercially available powders were homogenized by ball milling and consolidated at 900 °C using the spark plasma sintering (SPS) method. During cooling down, the specimens were subjected to isothermal holding at 350 or 250 °C for 1 h. After consolidation, all specimens were tested for density and hardness, whereas selected specimens were characterized by scanning electron microscopy (SEM) and flexural strength tests. The specimens made of BDCM50 powder (a mixture of the base and pre-alloyed powders in 50:50 proportion) shows excellent properties including σ_0.2_ = 1045 MPa in the three-point bending test and HV10 ≈ 380. Resistance to abrasive wear evaluated in both three-body and two-body conditions in the MWT abrasion test was estimated at $A_{i3}=18.1\pm 3.9$ μm/20 m and ${\;A}_{i2}=95.9\pm 11.8$ μm/20 m, respectively. A series of diamond-impregnated specimens (segments) was also produced and tested for wear rate on abrasive concrete. The potential graphitization of the diamond grits was investigated using Raman spectroscopy and X-ray diffraction. As a reference, both the base Fe-Mn-Cu-Sn-C and commercially available Co+20%WC alloy were used to compare selected properties of the investigated materials. It has been proved that heat-treated specimens made of the base mixture modified with pre-alloyed powders are characterized by increased hardness and resistance to abrasive wear. The BDCM50 matrix has a negligible effect on diamond graphitization and shows excellent field performance, which makes it a good potential substitute for replacing Co+20%WC in sintered diamond-impregnated tools.

## 1. Introduction

Over the last few years, systematic development in the concrete and stone floor grinding industry has been visible. The main reason for this is versatility. Concrete floors are perfect for utility, industrial or office spaces. Concrete has also inspired modern designers and interior architects. The combination of such values as aesthetics, durability, reduced construction costs and minimal impact on the environment led to the adoption of polished concrete as a new standard for flooring. One of the main services regularly provided by surface preparation companies is restoring old concrete floors to their original appearance. Typically, these floors are covered with tiles, carpeting, paint or epoxy coating. Therefore, it is particularly important to thoroughly remove the material from the floor and prepare an ideal base for further work. For this purpose, metal–diamond tools are used, the choice of which depends on the properties of the processed material (e.g., hardness, density, mineralogical composition, abrasive properties) and the processing conditions. When grinding the concrete and stone floors, two parameters of metal–diamond tools are important: tool performance, measured by the amount of polished surface as well as the pace of work, and tool wear, depending on the appropriate selection of diamond grit, matrix material and the type of material being processed. Maintaining the durability and quality of work of metal–diamond segments is related to the design of an appropriate structure and composition of the matrix. Due to diverse working conditions of metal–diamond tools, various types of materials are used as the matrix. Cobalt and its alloys (Co, Co+20%WC, Fe-Cu-Co-WC), due to their excellent retention properties and resistance to abrasive wear, are commonly used for the production of tools used for grinding concrete floors. However, in recent years, a new research trend towards the development of low-cobalt, or preferably cobalt-free, matrix materials has been observed [1,2,3]. Much work has been carried out using various manufacturing techniques to obtain the desired properties of the matrix material. Ye et al. [4] investigated a novel microwave hot-press sintering (MHPS) method for the improvement of mechanical properties of Fe-Cu-W-Sn matrix diamond tools. Li et al. [5] prepared Fe-based diamond composites with boron addition by pressureless infiltration sintering and showed that they can be successfully used as a replacement for the commonly used WC-based materials. The improvement of mechanical and tribological properties was also studied in [6,7,8,9,10]. Iron-based pre-alloyed powders were reinforced with oxides, carbides and their mixtures. Sun et al. [11] developed a new kind of matrix containing up to 2.0 vol.% of randomly dispersed SiC whiskers. As shown in [12,13,14], obtaining the desired mechanical and tribological properties is also possible through modification with alloy powders, which can effectively overcome the problems of component segregation, uneven element distribution, low alloying and diamond thermal damage. Shi et al. [15] investigated the influence of alloying on Fe-Cu based metal matrix composites. They showed that mechanical properties of Fe-Cu based metal matrix composites increased at lower Fe/Cu ratios but sharply decreased when Cu became the main component. The optimum Fe/Cu ratio for impregnated diamond bits was found to lie between 8:2 and 3:7. Hu et al. [16] studied the possibility of replacing the WC-containing drills with Fe-based and Cr-Fe-based ones. Luno-Bilbao et al. [17] studied the improvement in mechanical and tribological properties of matrix materials made from pre-alloyed Fe-Cu and Fe-Cu-Sn powders through the application of thermal treatment after sintering.

As a part of the research [18,19,20], a material with high hardness and resistance to abrasive wear was developed for sintered metal–diamond tools, which can successfully replace the commonly used Co+20%WC alloy. A further development of the Fe-Mn-Cu-Sn-C material was continued in [21], where the matrix was modified with SiC, Al_2_O_3_ and ZrO_2_ particles. Such matrices showing increased tribological properties are suitable for machining different types of abrasive concrete.

In the present study, an effort was made to obtain a tool material on the base of the Fe-Mn-Cu-Sn-C mixture characterized by better functional properties compared to the commonly used Co+20%WC alloy. Despite the positive effect of manganese on steel properties, it is well known that manganese has a high vapor pressure and high oxygen affinity that make materials containing Mn difficult to produce via the powder metallurgy route. The main goal was to partially substitute manganese content in the base alloy (Fe-Mn-Cu-Sn-C) with commercially available, relatively cheap iron-base pre-alloyed powders without losing high hardness and resistance to abrasive wear of the as-consolidated, heat-treated matrix.

## 2. Experimental Procedures

### 2.1. Materials

Pre-alloyed Astaloy CrM and Distaloy DC1 powders produced by Höganäs, Sweden, were used for the modification of the base Fe-Mn-Cu-Sn-C material. The base powder was made by milling a mixture of iron, ferromanganese, tin bronze and graphite powders. Detailed description of its production was presented in Refs. [18,19,20]. The chemical composition of the powders used in this work is given in Table 1.

Four powder compositions were prepared from the starting materials prior to consolidation. The powder preparation process consisted of the following stages:

- Milling a mixture of 49.7% Astaloy CrM, 49.7% Distaloy DC1 and 0.6% graphite in a planetary mill for 2 h, at 200 rpm (≈0.7 of the critical speed), in air. The milling took place in a WC-lined vial filled to 50% of its volume with 12 mm WC balls and the ball-to-powder weight ratio of 10:1. Thus obtained powder is henceforth referred to as DCM.
- Mixing the base Fe-Mn-Cu-Sn-C powder with 10, 30 and 50% of DCM for 2 h in a Turbula-type mixer.
- Ball milling of the mixtures in a planetary mill using conditions described above.

Designations of the investigated materials and their compositions are presented in Table 2.

### 2.2. Characterization

Prior to consolidation, all ball-milled powders were subjected to sieve analysis. The specimens were hot pressed at 900 °C for 10 min using the SPS technique. The thermal treatment of the specimens was carried out during the cooling down step. One batch of specimens was directly cooled to room temperature, whereas the other was held at either 350 or 250 °C for 1 h to investigate the effect of controlled cooling on hardness and density. The as-sintered densities were measured using the Archimedes method. The hardness of each specimen was determined on 5 indentations by the Vickers method under a load of 98.1 N, using the Future Tech FLC–50VX hardness tester. For 3-point bending tests, 29 × 7 × 4.5 mm specimens were produced in order to determine the transverse rupture strength (σ_TRS_), 0.2% offset yield strength (σ_0.2_) and strain at failure (ε_pl_).

Cylindrical (Ø11.3 × 5 mm) diamond-free and diamond-impregnated specimens were also produced for abrasive wear testing by admixing a proper amount of diamond particles with the tested powder mixtures and consolidating in the same conditions as their diamond-free counterparts. The diamond-containing specimens were also used for checking diamond graphitization. The methods for measuring wear resistance to both 3-body and 2-body abrasion were described in Refs. [22,23,24,25]. The degree of diamond graphitization was evaluated by a disperse Raman spectrometer DXR Microscope, Thermo Scientific, Waltham, MA, USA, equipped with an Olympus confocal microscope and a thermoelectrically cooled CCD detector. A solid-state Nd:YAG laser (wavelength 532 nm, maximum power 10 mW) was used as a sample excitation source. Measurements were performed with 900 lines/mm grating with a 50 µm pinhole aperture and 20× magnification objective using maximum laser power and 1 s acquisition time per scan. Five different diamond crystals, on 2–4 spots each, were measured with at least 10 repetitions. The degree of diamond graphitization was tested on sintered specimens as well as on samples additionally subjected to annealing at 900 °C for 10 min. To simulate harsh thermal conditions, measurements were repeated on specimens subjected to second annealing at 900 °C for 30 min. To prevent the matrix from oxidation, before each annealing process, the specimens were vacuum-sealed in glass capsules in the Department of Glass and Ceramics, UCT.

The microstructures were observed on polished and etched (2% Nital) cross-sections using an Axiovert 200 MAT light microscope from ZEISS and an FEI Versa 3D scanning electron microscope (field emission electron gun). In addition, X-ray diffraction (XRD) analyses were performed using a PANalytical X’Pert PRO diffractometer equipped with a Cu radiation source (λ_Cu_ = 1.5406 Å).

Wear tests under industrial conditions were carried out on 40 × 10 × 10 mm diamond-impregnated specimens, which were produced in a similar manner. High-quality synthetic diamond grits with 60/80 US mesh at concentration c = 20 (5 vol.%) were used. Obtained segments were brazed to steel plates with the induction method using an Ag45Sn solder to produce grinding wheels. The wear test (grinding process) was performed on 1500 × 1500 × 100 mm slabs made of concrete. To vary grinding conditions, three types of concrete differing in density and compressive strength were used: C8/10, C12/15 and C25/30. The grinding process was utilized using HTC 420 vs. the concrete floor grinder using 65 kgf force and 560 rpm. Before wear tests, a few run-in cycles were carried out for tool conditioning purposes. The diamond-impregnated segments were tested for wear rate during 30 min periods. Before and after the wear test, the segments were cleaned and carefully measured in order to calculate the loss of volume. For reference, the base (Fe-Mn-Cu-Sn-C) and commercial Co+20%WC powder mixtures were used as a matrix to produce diamond-free and diamond-impregnated specimens using the same consolidation procedure.

## 3. Results and Discussion

### 3.1. Powder Characterization

**Figure 1:**
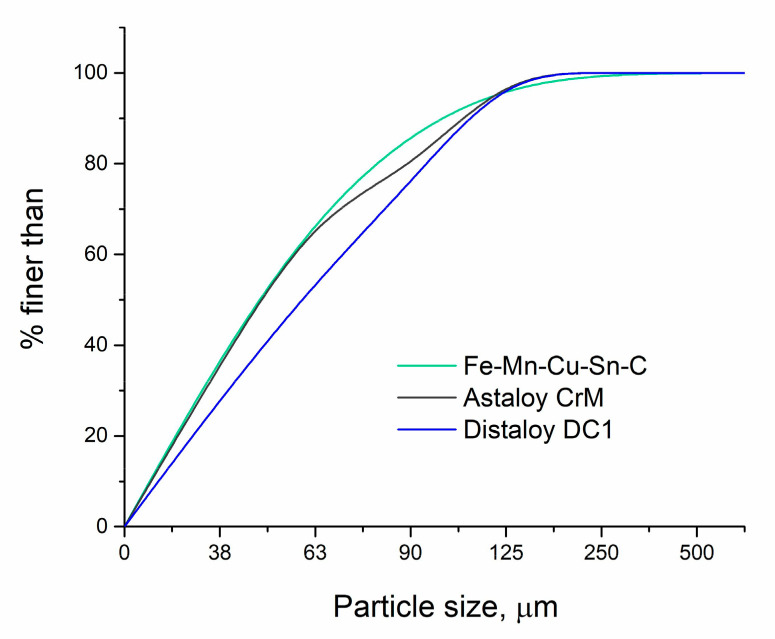
Cumulative curves of particle size distribution.

The results of sieve analysis are presented in the form of a cumulative curves in Figure 1. In order to determine the shape and confirm the particle size of the powders, they were subjected to morphology studies using scanning electron microscopy.

**Figure 2:**
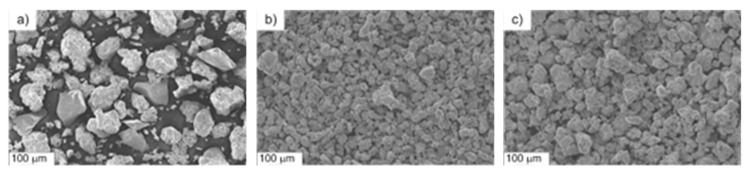
Morphology of the raw powders (SEM): Fe-Mn-Cu-Sn-C (**a**); Astaloy CrM (**b**); Distaloy DC1 (**c**).

The bulk properties of the experimental powder are presented in Table 3, whereas the powder particle morphology is shown in Figure 2.

The base Fe-Mn-Cu-Sn-C powder is characterized by irregular shape. The Astaloy CrM and Distaloy DC1 particles have a spongy shape. These powders show considerable homogeneity of particle size in comparison to the ball-milled Fe-Mn-Cu-Sn-C powder.

### 3.2. Composite Characterization

#### 3.2.1. Density and Hardness Measurements

**Figure 3:**
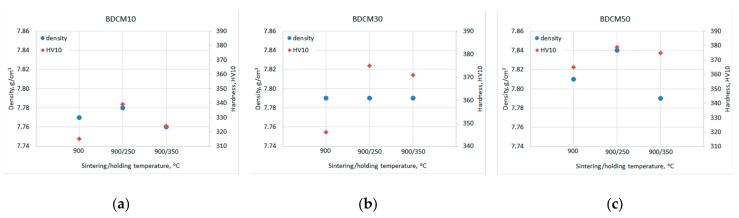
The effect of cooling conditions on relative density and hardness of the as-sintered materials BDCM10 (**a**); BDCM30 (**b**) and BDCM50 (**c**); 900—direct cooling, 900/250—holding at 250 °C for 1 h, 900/350—holding at 350 °C for 1 h.

The as-sintered specimens were tested for density and hardness (Table 4) in order to examine the effect of different cooling conditions—Figure 3a–c. Based on the obtained data, the optimal consolidation conditions were chosen for each material.

All tested materials show a similar effect of cooling on hardness for which maxima were recorded after sintering at 900 °C and subsequently cooling with holding at 250 °C for 1 h. The highest hardness (HV10 ≈ 380) showed specimens containing 50 wt.% of DCM powder. Therefore, this material was subjected to bending and tribological studies. The obtained results were compared both with the base Fe-Mn-Cu-Sn-C and Co+20%WC materials.

#### 3.2.2. Flexural Strength

**Figure 4:**
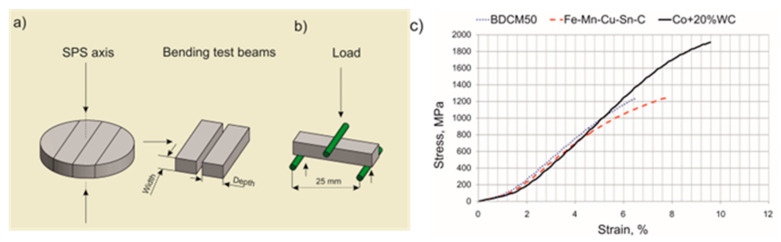
A schematic illustration of the three-point bending test: preparation of test beams (**a**); test fixture (**b**) and recorded stress–strain curves (**c**).

The three-point bending test was performed on nonstandard specimens to determine the transverse rupture strength (σ_TRS_), 0.2% offset yield strength (σ_0.2_) and strain at failure (ε_pl_). The testing procedure and stress–strain behavior of the selected materials are presented in Figure 4.

The results (Table 4) indicate that the sintered BDCM50 material was characterized by higher bending strength (σ_TRS_ = 1239 ± 70 MPa) and yield strength (σ_0.2_ = 1045 ± 35 MPa) but lower strain than the base Fe-Mn-Cu-Sn-C material (ε_pl_ = 1.38 ± 0.4%, ε_pl_ = 2.62 ± 0.8%, respectively). The higher strength of the BDCM50 material is associated with the isothermal phase transformation that occurred during controlled cooling, which resulted in the formation of lower bainite which was confirmed by microstructural examinations. For the commercial material, the tested values of σ_TRS_ and σ_0.2_ are much higher, and they are the result of the hard WC reinforcing phase.

#### 3.2.3. Wear Testing of Non-Diamond Specimens

**Figure 5:**
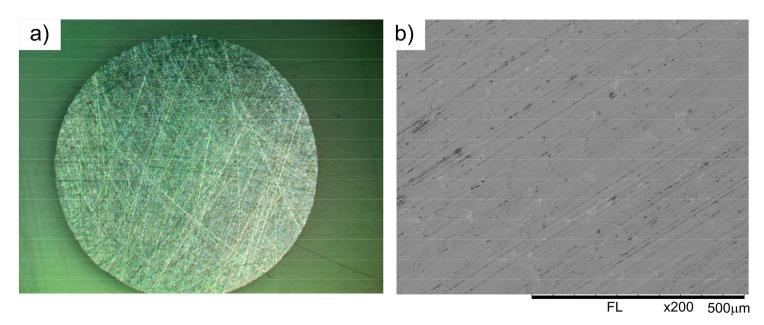
Worn surface of the BDCM50 specimen: LM (**a**); SEM (**b**).

The tribological properties of sintered metal–diamond segments are particularly important for their application. Depending on the workpiece being treated, there are different requirements for matrix materials. For example, machining of highly abrasive workpieces imposes high wear resistance on the matrix. Hence, the abrasive wear test is crucial to predict a potential application of the tested materials. The MWT method [22,23,24,25] was used to compare materials used for the matrix in sintered diamond-impregnated tools for processing of concrete (also reinforced) and natural stones. The abrasive wear index ($A_{i}$), representing the average loss of height of three test pieces per 20 m sliding distance, was calculated for three-body abrasion using a loose quartz sand finer than 200 μm ($A_{i\left(3\right)}$) and two-body abrasion on #220 SiC grinding paper ($A_{i\left(2\right)}$). The results are summarized in Table 4, whereas the worn surfaces are shown in Figure 5. The best results were obtained for specimens made of BDCM50 containing pre-alloyed powders ($A_{i3}=18.1\pm 3.9$ μm/20 m, ${\;A}_{i2}=95.9\pm 11.8$ μm/20 m). The increased resistance to wear is probably the result of phase transformation during controlled cooling, leading to lower bainite formation, which has high hardness and abrasion resistance. The results show that by proper modification of chemical composition of the matrix and consolidation process, it is possible to produce materials characterized by reduced wear rate.

#### 3.2.4. Microscopic Observation

**Figure 6:**
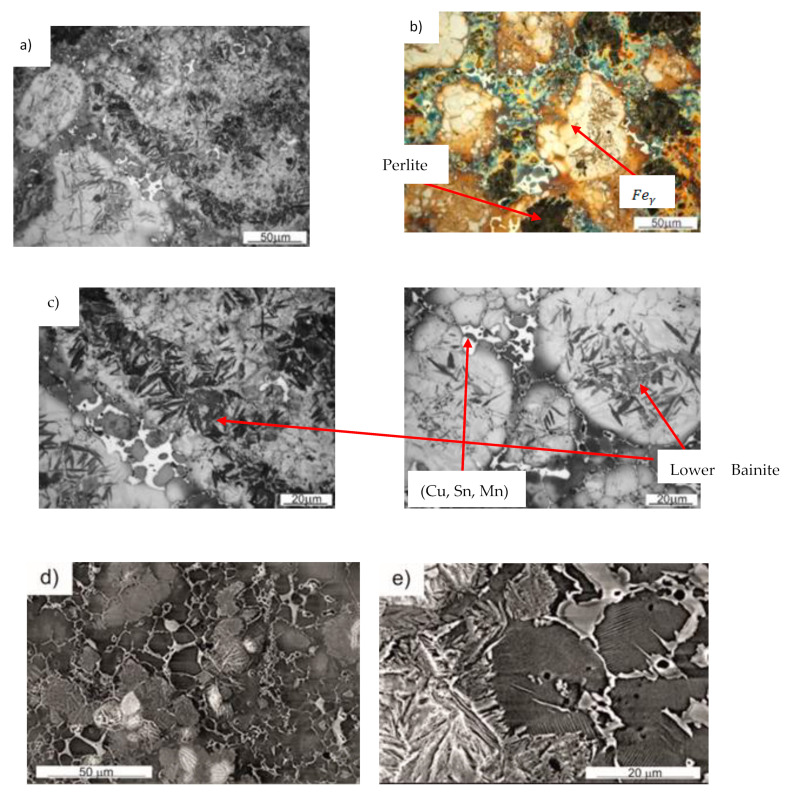

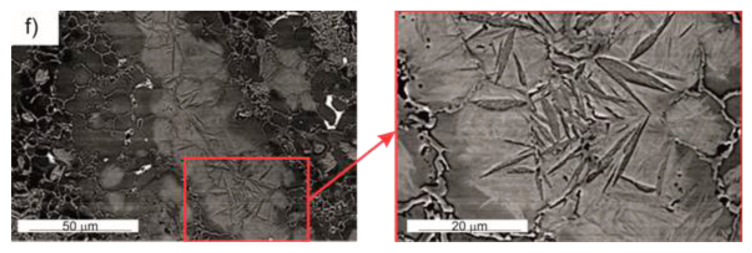
Microstructure of BDCM50 after isothermal holding at 250 °C for 1 h: LM (**a**–**c**); SEM (**d**–**f**).

**Figure 7:**
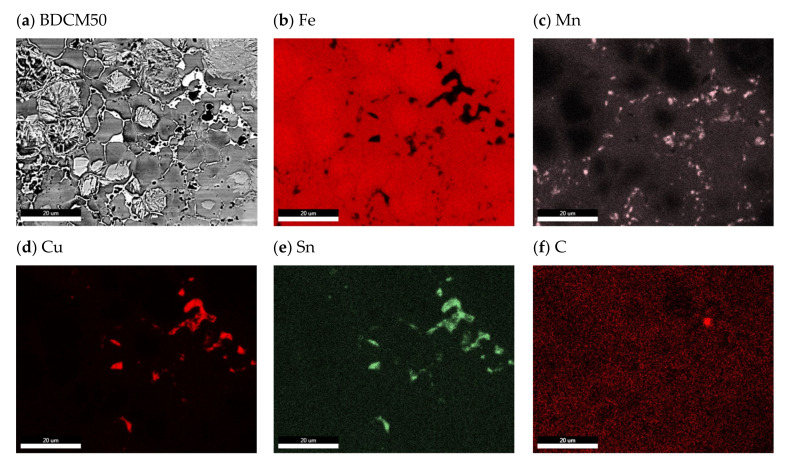
EDS maps of element distribution after isothermal holding at 250 °C for 1 h.

The microstructure of the BDCM50 material after isothermal holding at 250 °C for 1 h is heterogeneous, with austenitic areas enriched with Mn, lower bainite, perlite, ferrite and tin bronze. Figure 6 shows selected images of the microstructure, whereas the surface distribution of elements (EDS maps) is presented in Figure 7. It is easy to distinguish microstructural constituents. The bronze areas are present in Cu, Sn and Mn. The lower bainite seen in Figure 6c,f has a plate (lath) morphology in which the carbides lie at angle of approximately 55° to the plate’s axis. It is worth underlining that there is no brittle martensite. In diamond-impregnated tools, a brittle matrix is undesirable. The brittleness of the matrix is one of the factors responsible for an extensive diamond particle loss during cutting/machining of a workpiece, which significantly shortens the tool life.

#### 3.2.5. Raman Spectroscopy

**Figure 8:**
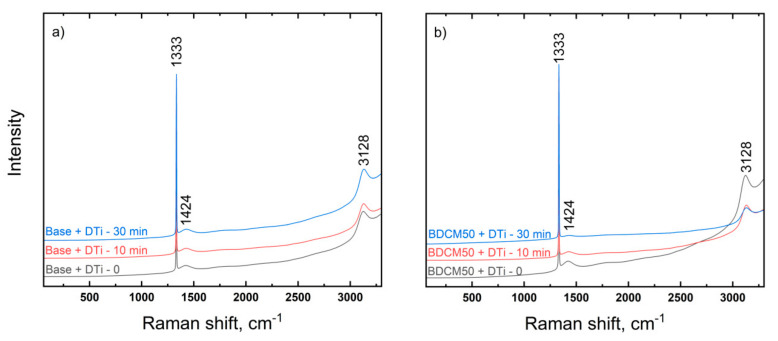
Average Raman spectra of diamonds at room temperature and after annealing for base (Fe-Mn-Cu-Sn-C) (**a**) and BDCM50 (**b**) matrices. Spectra are offset for clarity.

**Figure 9:**
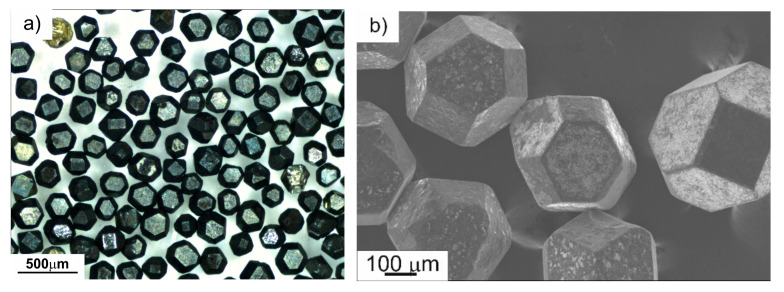
MBS-970 grade diamond crystals after extraction from the BDCM50 segment, LM (**a**) and SEM (**b**).

The diamond graphitization test was performed on Fe-Mn-Cu-Sn-C and BDCM50 segments. The average Raman spectra for all variants (after SPS**,** after annealing at 900 °C for 10 min and after second annealing at 900 °C for 30 min) are shown in Figure 8. Graphitization manifests itself on the Raman spectrum via bands at ~1580 cm^−1^, which belong to sp^2^ hybridized carbon atoms. The recorded Raman spectra show no evidence of diamond graphitization on the tested specimens. The most intense peak at 1332 cm^−1^ is associated with crystalline diamond. Less intensive peaks around 1420 and 3125 cm^−1^ are probably attributed to fluorescence bands of nitrogen-vacancy centers in the diamond lattice. Thus, the annealing process (even for a prolonged time) has negligible effect on quality of diamond particles embedded in the investigated materials. Microscopic examinations of diamond crystals after extraction from metal–diamond segments showed the preservation of the original shape, flat crystal facets and sharp edges (Figure 9). The blackened surface of the particles is probably caused by some pyrolyzed impurities deposition during sintering.

#### 3.2.6. X-ray Diffraction

**Figure 10:**
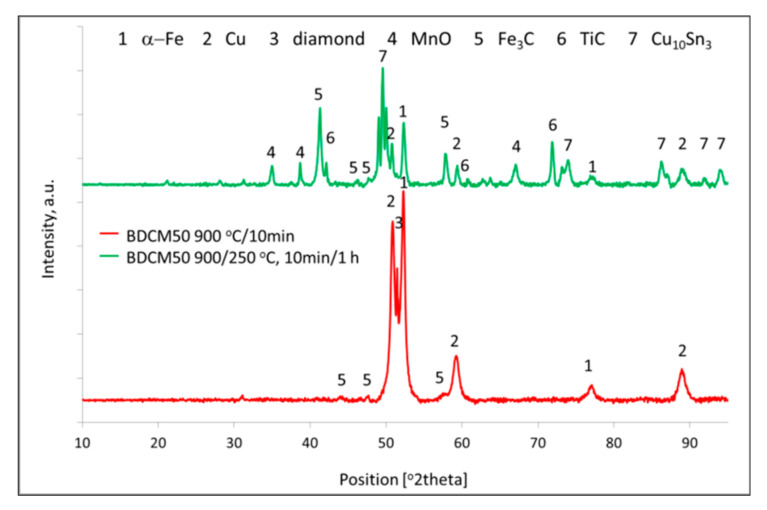
XRD analysis of BDCM50 specimens. sintered by SPS at: 900 °C/10 min. and sintered and held at 250 °C for 1 h. Spectra are offset for clarity.

The X-ray diffraction analysis of BDCM50 materials sintered at 900 °C for 10 min and sintered with isothermal holding at 250 °C for 1 h is presented in Figure 10. A higher Fe_3_C peak was recorded on the specimen after controlled cooling. This may suggest that bainitic phase transformation or precipitation of carbides take place. A direct cooling from 900 °C to room temperature resulted in a hardening effect, probably due to martensitic transformation; thus, a very small Fe_3_C peak was recorded. On the other hand, after isothermal cooling, instead of martensite, a lower, very fine bainite is probably formed, which significantly increases the wear resistance of the material. In case of the specimens continuously cooled down after sintering, the wear resistance is lower because of the change in wear mechanism from plastic deformation to chipping of hard and brittle martensite. In addition to Raman spectroscopy, X-ray patterns recorded for both materials show no traces of diamond graphitization.

#### 3.2.7. Wear Testing of Diamond-Impregnated Specimens

**Figure 11:**
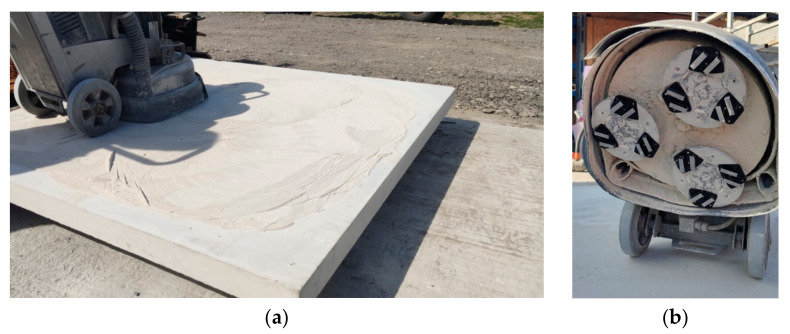
Stand for the grinding test (**a**) and assembly of diamond-impregnated segments (**b**).

**Figure 12:**
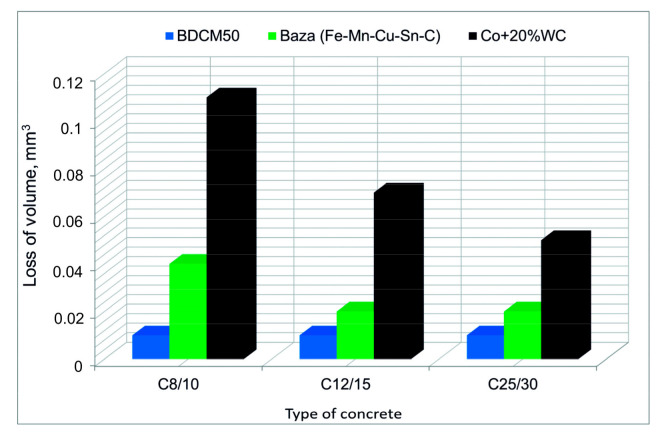
Loss of volume of metal–diamond segments depending on the type of concrete.

The metal–diamond tools used for the field performance tests based on grinding of concrete surfaces are shown in Figure 11. The results of the study are presented in Figure 12. The obtained data confirmed that by addition of pre-alloyed powders to the base matrix (Fe-Mn-Cu-Sn-C), it is possible to significantly increase the tool life. The recorded volume loss for this material of ΔV < 0.02 mm^3^ was the lowest for all investigated matrices, even those based on Co+20%WC. It is worth underlining that segments with Co+20%WC matrix were characterized by the highest abrasive wear, which is in a very good agreement with the MWT results. Thus, in order to produce an efficient tool characterized by a long tool life, a proper balance between hardness and elastic properties must be achieved. From the results obtained in the grinding test, it is also evident that harsh conditions during grinding of very abrasive C8/10 concrete generate large amount of sludge, which leads to intensive wear of the tested materials.

## 4. Conclusions

This study presents the sintering behaviors, microstructures, physical, mechanical and tribological properties of iron-based matrix composites modified with pre-alloyed powders. It has been shown that by using the SPS method it is possible to obtain virtually pore-free materials. Specimens modified with pre-alloyed powders are characterized by increased hardness and resistance to abrasive wear. It has been proved that it is possible to partially substitute the base powder (Fe-Mn-Cu-Sn-C) with commercially available pre-alloyed powders which are intended for structural parts. By ball milling powder mixtures made of cheaper powders, matrix materials can be obtained for use in the powder metallurgy diamond tools. It is worth noting that even isothermal holding at 250 or 350 °C or prolonged annealing at 900 °C of segments made of investigated materials has a negligible effect on diamond graphitization, and tools show excellent field performance. In view of the obtained results, the BDCM50 matrix has high potential for commercial application for replacing Co+20%WC in sintered diamond-impregnated tools.

## Figures and Tables

**Table 1:** Chemical compositions of the experimental powders.

Powder	Chemical Composition (wt.%) ^1^							
	Fe	Mn	Cu	Sn	C	Cr	Mo	Ni
Base (Fe-Mn-Cu-Sn-C)	bal.	12	6.4	1.6	0.64			
Astaloy CrM	bal.				0.006	2.94	0.52	
Distaloy DC1	bal.		0.04		0.004		1.51	1.98

^1^ Chemical composition of Astaloy CrM and Distaloy DC1 powders taken from certificates.

**Table 2:** Composition of the investigated materials.

Designation	Powder Composition (wt.%)		Chemical Composition (wt.%)							
	DCM	Fe-Mn-Cu-Sn-C	Fe	Mn	Cu	Sn	C	Cr	Mo	Ni
BDCM10	10	90	81.0	10.8	5.8	1.4	0.6	0.1	0.1	0.1
BDCM30	30	70	84.3	8.4	4.5	1.1	0.6	0.4	0.3	0.3
BDCM50	50	50	87.6	6.0	3.2	0.8	0.6	0.7	0.5	0.5

**Table 3:** Physical properties of the experimental powders.

Powder	Apparent Density (g/cm^3^)	Mean Particle Size ^2^, µm
Base (Fe-Mn-Cu-Sn-C)	3.57	86
Astaloy CrM	2.81	91
Distaloy DC1	3.05	98

^2^ Weighted arithmetic mean.

**Table 4:** Density, hardness, abrasion resistance indices and bending properties of the tested materials.

Material	Density ^(1)^ (g cm^−3^)	HV10 ^(1)^	$A_{i\left(3\right)}$ ^(1)^ (µm/20 m)	$A_{i\left(2\right)}$ ^(1)^ (µm/20 m)	Three-Point Bending Test ^(1)^		
					σ_TRS_ (MPa)	σ_0.2_ (MPa)	ε_pl_ (%)
BDCM50	7.82 ± 0.01	379 ± 23	18.1 ± 3.9	95.9 ± 11.8	1239 ± 70	1045 ± 35	1.38 ± 0.4
Base (Fe-Mn-Cu-Sn-C)	7.75 ± 0.01	299 ± 7	24.6 ± 2.7	138.7 ± 1.2	1223 ± 103	924 ± 29	2.62 ± 0.8
Co+20%WC	9.25 ± 0.01	374 ± 17	48.8 ± 5.9	177.1 ± 9.2	1929 ± 162	1737 ± 79	1.84 ± 0.8

^(1)^—scatter intervals estimated at 90% confidence level.

## Data Availability

The data presented in this study are available on request from the corresponding author.
